# Role of Selenoprotein F in Protein Folding and Secretion: Potential Involvement in Human Disease

**DOI:** 10.3390/nu10111619

**Published:** 2018-11-02

**Authors:** Bingyu Ren, Min Liu, Jiazuan Ni, Jing Tian

**Affiliations:** 1 Shenzhen Key Laboratory of Marine Biotechnology and Ecology, Department of Marine Biology, Shenzhen University, Shenzhen 518060, China; renbingyu89@foxmail.com (B.R.); 2170257211@email.szu.edu.cn (M.L.); 2 Changchun Institute of Applied Chemistry, Chinese Academy of Sciences, Changchun 130022, China; 3 Shenzhen Engineering Laboratory for Marine Algal Biotechnology, College of Life Sciences and Oceanography, Shenzhen University, Shenzhen 518060, China; jzni@szu.edu.cn; 4 Shenzhen Key Laboratory of Microbial Genetic Engineering, College of Life Sciences and Oceanography, Shenzhen University, Shenzhen 518060, China

**Keywords:** selenium, selenoprotein F, thiol–disulfide oxidoreductase, endoplasmic reticulum stress, protein folding quality control, single nucleotide polymorphisms

## Abstract

Selenoproteins form a group of proteins of which its members contain at least one selenocysteine, and most of them serve oxidoreductase functions. Selenoprotein F (SELENOF), one of the 25 currently identified selenoproteins, is located in the endoplasmic reticulum (ER) organelle and is abundantly expressed in many tissues. It is regulated according to its selenium status, as well as by cell stress conditions. SELENOF may be functionally linked to protein folding and the secretion process in the ER. Several studies have reported positive associations between *SELENOF* genetic variations and several types of cancer. Also, altered expression levels of SELENOF have been found in cancer cases and neurodegenerative diseases. In this review, we summarize the current understanding of the structure, expression, and potential function of SELENOF and discuss its possible relation with various pathological processes.

## 1. Background

As a trace element, selenium is nutritionally essential for mammals. A lot of evidence has revealed the role of selenium in preventing cancers and maintaining the proper function of the thyroid, the immune system, and reproduction [1]. Selenium deficiency is connected to cardiovascular, aging-associated, immune, and brain diseases [2,3,4,5]. Nevertheless, selenium has been found to have a two-sided effect on human health, depending on its concentration. Excessive selenium can be toxic, and a tolerable upper intake level of selenium has been established at 300 μg/person per day in the EU [6]. Also, a U-shaped association has been noted between selenium status and its health effects [7].

The functions of selenium are believed to be enabled by the 21st amino acid, selenocysteine (Sec, U, Se-Cys) [8]. Sec is encoded by the TGA codon, which is recognized as a stop codon in general cases. Specific complex machinery is required to co-translationally incorporate Sec into the growing polypeptide chain, including selenocysteine insertion (SECIS) elements in the selenoprotein mRNA secondary structures and trans-acting factors that interact with SECIS elements [9].

Proteins containing Sec are grouped into the so-called selenoproteins, which function as regulators, structural proteins, essential antioxidant enzymes, and others [10]. The protective effect of selenium is achieved through the regulation of the expression of selenoproteins. Twenty-five selenoproteins have been discovered in the human proteome [11]. Selenoprotein F (SELENOF), the new name according to the selenoprotein gene nomenclature, was initially named the 15-kDa selenoprotein (Sep15) [12]. It was first characterized in human T-cells by Gladyshev in 1998. A selenocysteine residue was found in the polypeptide sequences of SELENOF, together with a SECIS element that was located in the 3′-UTR of its mRNA, thus meeting the criteria of a selenoprotein [13]. Though the function of SELENOF is only partially established so far, its potential roles in protein folding and secretion have been indicated. Here, we summarize the available knowledge relating to the structure, distribution, and regulation of SELENOF, explore the role of SELENOF in protein redox quality control, and discuss the possible associations between *SELENOF* genetic polymorphisms/dysregulation and pathologies.

## 2. The Cellular Localization and Structure of SELENOF

The human *SELENOF* gene is located on chromosome 1p31 [14]. The gene product SELENOF is a protein that is localized in the endoplasmic reticulum (ER) with a molecular mass that is close to 15 kDa. It has been found in various species, from green algae to humans. Alignment results have revealed that its sequence is highly conserved and shares 31% similarity with another ER selenoprotein, Selenoprotein M [15].

According to the relative location of Sec in the polypeptide chain, selenoproteins can be divided into two groups. One group contains Sec close to the C-terminal, and the other group has Sec in the N-terminal part, which has a thioredoxin (Trx)-like structure in most cases [11,16]. SELENOF belongs to the latter group. Structural studies have revealed an ER signal peptide at its N-terminus, which directs the newly synthesized SELENOF to the ER where it will be cleaved into its mature form. Though no typical ER-resident peptide was found in the SELENOF sequence, it is maintained in ER through a tight association with another protein, UDP-glucose:glycoprotein glucosyltransferase (UGGT), through a cysteine-rich domain [17]. An atypical C*x*U motif, in which Cys is separated from Sec by only one amino acid, is located in the Trx-like domain at the C-terminus [18]. Since Sec is usually present as an active-site residue and the C*x*U motif of SELENOF is redox-active and surface accessible, catalytic activity can be expected from SELENOF. The equilibrium redox potential of the fruit fly SELENOF protein has been measured to be −225 mV, which is right between the redox potentials of Trx and protein disulfide isomerase (PDI), suggesting that SELENOF is capable of reducing and/or isomerizing the disulfide bonds of proteins [18].

## 3. The Expression and Regulation of SELENOF

The ER is the major cellular component for secreted protein manufacture and glycosylation initiation. As an ER protein, SELENOF can be detected in a wide variety of human tissues. It is notably expressed in tissues with secretory functions, such as thyroid, liver, and kidney; in reproductive organs, such as prostate and testis [14]; and in abundance in brain regions like the hippocampus and cerebellum [19]. In silicopredictions of the human *SELENOF* gene have found a typical CpG island (regions with a high frequency of CpG siteswhere a cytosine nucleotide is followed by a guanine nucleotide), two putative metal response elements (MREs), and four putativenuclear factor kappa-light-chain-enhancer of activated B cells (NFκB) binding sites located upstream of its transcription start site [20] which may mediate the tissue-specific transcriptional expression of SELENOF. Our research group has confirmed that NFκB mediates the transcriptional regulation of SELENOF expression in HEK293T cells [21]. Interestingly, MREs are features of the binding sequences of metal regulatory transcription factors, a class of transcription factors that regulate the transcriptional response to heavy metal exposure, oxidative stress, and hypoxia. The possibility that metal regulatory transcription factors regulate SELENOF expression needs to be further clarified.

Hierarchical principles of selenoprotein expression in response to selenium have been noted by researchers using different tissues or cell models [22,23]. Generally, selenoproteins have been classified into two groups: “house-keeping” selenoproteins that are resistant to selenium changes, and “stress-regulated” selenoproteins that are sensitive to selenium changes [24]. Ina recently published study, SELENOF was assigned to the selenium-sensitive “stress-regulated” group [25]. The expression of SELENOF has been reported to be regulated by selenium bothin vitro and in vivo. In primary mouse colon cells, both selenite and methylseleninic acid (MeSeA) increased SELENOF protein levels in a dose-dependent manner, while the very same concentrations of Se-methyl selenocysteine (SeMeSeCys) and selenomethionine (SeMet) had no effect. Notably, the mRNA levels of SELENOF remained unaffected by all four of these selenium compounds, indicating that the increase in SELENOF protein levels may result fromthe translation of still-present mRNA [26]. In chickens, dietary selenium deficiency led to a decrease inSELENOF mRNA [27]. In the liver of selenium-supplemented growing lambs, SELENOF mRNA was found to be upregulated [28]. Furthermore, selenium can prevent the downregulation of SELENOF mRNA which is caused by toxicants or oxidative stress [29,30,31].

Beyond that, the expression of SELENOF is also connected with the state of the ER. Different changing tendencies of SELENOF protein levels have been observed in adaptive and acute ER stress conditions [19]. The pharmacological ER stress inducers Tunicamycin and Brefeldin A increased SELENOF during a 24 h treatment, while Thapsigargin and Dithiothreitol (DTT) stimulated the rapid degradation of SELENOF [19]. Notably, both Tunicamycin and Brefeldin A trigger ER stress by interfering with protein synthesis, which indicates the involvement of SELENOF during this process.

## 4. Role of SELENOF in Redox Protein Quality Control

It has been recognized by researchers that the antioxidative and stress-relieving effects of selenium are mainly achieved by its incorporation into the selenoproteins with oxidoreductase functions [32]. During adaptive ER stress, SELENOF has been found to be upregulated with a series of protective cellular actions, such as the Unfolded Protein Response [19]. Thus, the protective function of SELENOF can be assumed to occur during the selenium-mediated antioxidative process and under ER stress conditions.

Seven selenoproteins have been identified as residents of the ER, including Iodothyronine deiodinase 2 (DIO2) and selenoproteins F, K, M, N, S, and T. Though the functions of these ER-resident selenoproteins have not been fully characterized, evidence has indicated their involvement in several ER processes, such as maintaining redox or calcium homeostasis, quality control, and the endoplasmic-reticulum-associated protein degradation (ERAD) machinery [33]. As SELENOF’s binding partner, UGGT participates in ER protein folding quality control by reglucosylating misfolded glycoproteins [34]. SELENOF has been found to form a heterodimeric complex with both UGGT isoforms and markedly enhance their glucosyltransferase activity [35]. The UGGT1 knockout is embryo lethal, revealing that it is indispensable for embryogenesis and maintaining tissue function [36]. Knockout studies of UGGT1 in cultured cells have demonstrated its effect on protein solubility and secretion rates [37,38]. Since structural studies have indicated that SELENOF may exhibit thiol–disulfide oxidoreductase activity, it may also play a role in the glycoprotein folding process of misfolded proteins that are recognized by UGGT as its potential substrates [15], suggesting that SELENOF deficiency possibly results in similar outcomes to the UGGT knockout.

We summarize in Table 1 the main contents of the SELENOF knockdown or knockout studies that have been reported so far. SELENOF knockout mice are viable and fertile, so observation of abnormal protein remodeling or protein secretion have been reported in SELENOF deficiency cases. In the latest work on knockout mice, a nonfunctional increase in the secretion of the disulfide-rich glycoprotein Immunoglobulin M (IgM) and a delay of ER–Golgi protein transportation were detected. Based on this result, together with previous reports, a gatekeeper function of SELENOF in the redox quality control process of glycoprotein secretion has been proposed. Recently, we identified Retinol dehydrogenase 11 (RDH11) as an interacting protein for SELENOF. SELENOF overexpression caused a decrease in exogenous RDH11 retinal reductase activity, suggesting that SELENOF may also affect protein enzyme activities [39].

## 5. Associations of SELENOF and Disease Pathologies

Connections have been noted between selenium status and many disease progressions, such as various cancers, immune system diseases, and neurodegenerative diseases [32,49,50]. Though supplementation with selenium has obtained some beneficial effects during these cases, current findings are not conclusive enough and are sometimes inconsistent. For example, both the Selenium and Vitamin E Cancer Prevention Trial (SELECT) and a Phase III trial of selenium have failed to confirm the effective role of selenium in prostate cancer prevention from the Nutritional Prevention of Cancer Trial (NPC) results [51]. The disparate results of these trials may be explained by the U-shaped relationship between selenium status and protection from cancer [7].

Besides selenium status, attention has also been paid to the possible roles of selenoproteins in these pathologies. Evidence for the effect of SELENOF deficiency on several different cellular events has been reported, together with the abundant expression of SELENOF in various organs, which we reviewed above, suggesting that SELENOF could be associated with multiple pathological processes in different tissues.

### 5.1. SELENOF Gene Polymorphisms and Pathologies

*SELENOF* genetic variations have been found to be associated with cancer etiology. Two single-nucleotide polymorphisms (SNPs), rs5845 and rs5859, have been identified in its 3′-untranslated region within the selenocysteine insertion sequence-like structures [14]. A significant association between the rs5845 T allele variant and elevated breast cancer risk in African-American women has been observed [14]. However, the same genotype shows no association with breast cancer risk or clinicopathological parameters in Caucasian women [52]. A significant difference in genotype distribution of the other site of polymorphism, rs5859, has been found between breast cancer patients and controls in the population of Iran [53]. In smoking individuals, the rs5859 AA genotype may still benefit from selenium when its plasma concentration is higher than 80 ng/mL, whereas in those with the GG or GA genotype, a relatively high selenium status could increase the risk of lung cancer [54]. Both the rs5845 and rs5859 sites with minor A and T alleles are associated with increased risk of male rectal cancer in the Korean population, which suggests that the effect of the two SNPs on cancer may be gender-dependent [55]. Another four common SNPs within the *SELENOF* gene—rs479341, rs527281, rs561104, and rs1407131—have also been identified. Though the polymorphisms rs479341, rs1407131, and rs561104 are not significantly associated with prostate cancer risk, these SNPs are significantly associated with prostate cancer mortality [56].

In addition, connections have been found between *SELENOF* gene polymorphisms and other diseases or functions. In Kashin–Beck disease, the minor Aallele frequency of rs5859 is statistically significantly higher [57]. This AA genotype at the rs5859 site is also associated with a shorter time of progression to AIDS compared with GG homozygotes [58]. In volunteers that aremore than 50 years old, rs5845 C allele variants received higher verbal learning memory scores than T allele variants [59]. The polymorphic sequences of rs5845 and rs5859 can alter the Sec incorporation efficiency of SELENOF SECIS elements [14], indicating that *SELENOF* genetic polymorphism may lead to different SELENOF protein expression levels in response to selenium, which might contribute to these disease pathologies.

### 5.2. SELENOF Dysregulation in Pathologies

Dysregulation of SELENOF at mRNA levels has also been noted in several disease cases or pathological models. SELENOF was found to be upregulated in two hepatocellular carcinoma cell lines, HepG2 and Huh7, compared with normal human hepatocytes [60]. On the contrary, it was downregulated in almost 60% of the malignant mesothelioma cell lines and tumor specimens compared with normal mesothelial cells [40]. Also, the expression of SELENOF mRNA was downregulated in the hippocampus and substantia nigra brain regions of a Parkinson’s mouse model [61] and in the leukocytes of bladder cancer patients [62].

## 6. Conclusions and Perspectives

Sec is encoded by the genetic code UGA, which is recognized as a stop codon in general cases. It is difficult to overexpress the wild-type selenoproteins in cell or animal models, which limits the approaches for SELENOF functional studies. Still, the understanding of SELENOF’s unique structure, subcellular localizations, binding partner, tissue distribution, and regulation will give us some clues about its potential function. A series of deficiency studies have revealed its effect on protein secretion, supporting the speculation that SELENOF plays a role in the redox protein folding process in the ER.

**Figure 1:**
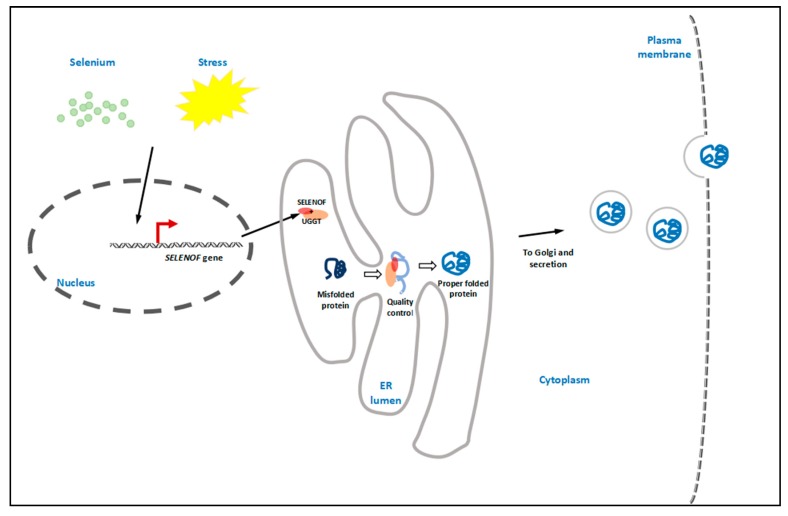
Graphical conclusion. As an endoplasmic reticulum (ER) selenoprotein, SELENOF can be regulated by both selenium status and ER stress. It forms a 1:1 tight complex with UDP-glucose:glycoprotein glucosyltransferase (UGGT) in the ER lumen, thus enhancing the enzymatic activity of UGGT. SELENOF may be involved in glycoprotein folding quality control by rearranging or reducing the disulfide bonds of UGGT-recognized misfolded proteins. The properly folded proteins are then packed into vesicles, followed by Golgi transportation and secretion.

As shown in the concluding Figure 1, as an ER selenoprotein, SELENOF can be regulated by both selenium status and ER stress. SELENOF forms a complex with UGGT and is involved in glycoprotein folding quality control. The newly synthesized and folded proteins are packed into vesicles, followed by Golgi transportation and secretion.

Accordingly, an increasing number of studies have linked *SELENOF* gene polymorphisms and SELENOF dysregulation to various diseases, including several types of cancer, AIDS, and neurodegeneration, which reveals the importance of SELENOF’s physiological functions. Currently, the mechanisms underlying SELENOF’s associations with numerous pathological states are not fully understood, and additional work is required to confirm SELENOF’s role in protein quality control. Recently, we applied a biotin labeling method to the screening and identification of SELENOF’s potential substrates (data unpublished) which may help us to better understand SELENOF’s involvement in various cellular processes. Promising results have been reported in cases applying selenium compounds to cancer and neurodegeneration treatment. Since SELENOF is selenium-sensitive, it may be a noteworthy potential target during these pathologies.

## Figures and Tables

**Table 1:** Summarization of the SELENOF (Selenoprotein F) knockdown and knockout studies.

No.	Models	Methods	Phenotypes	Pathways or Biological Processes that Were Possibly Involved	Published Year
1	Mouse malignant mesothelioma (MM) cells	siRNA	The expression of SELENOF was downregulated in most MM cases. Differential effects of selenium on MM cell growth were associated with genotype and expression of SELENOF.	The selenium-induced MM cell apoptosis was increased in cells that were transfected with wild-type SELENOF, however not with the 1125A variant. SELENOF siRNA inhibition made the sensitive MM cells more resistant to selenium [40].	2004
2	Mouse CT26 colon cancer cells and Lewis lung carcinoma (LLC1) lung cancer cells	Stably transfecte-d shRNA	Tumorigenicity and metastasis inhibition together with G2/M cell cycle arrest in colon cancer cells; no effect on lung cancer cells.	Genes significantly affected by SELENOF downregulation belonged to cancer, cellular growth, and proliferation biological processes [41].	2010
3	Mouse	Knockout	Mice were viable and fertile, with normal brain morphology and no activation of endoplasmic reticulum(ER) stress. The oxidative stress was elevated in the livers, and prominent nuclear cataracts were developing at an early age.	SELENOF mRNA level was progressively elevated in the lens during mouse development. An improper folding status of lens proteins was possibly caused by SELENOF deficiency [42].	2011
4	Mouse	Knockout	Protected mice against chemically induced colon cancer by inhibiting aberrant crypt formation.	SELENOF knockout resulted in upregulation of Guanylate binding protein-1 mRNA and protein expression and a higher level of interferon-γ in plasma [43].	2012
5	Chang liver cells	Doxycycli-ne-inducible shRNA	Actin and Tubulin cytoskeleton protein remodeling and non-apoptotic membrane blebbing.	SELENOF knockdown induced Ras homolog gene family, member A (RhoA) activation and phosphorylation of myosin phosphatase target subunit 1, and the remodeling of F-actin and α-tubulin was different from typical apoptotic blebbing cells [44].	2015
6	Chang liver cells	Doxycycli-ne-inducible shRNA	Cell proliferation and motility inhibition together with G1 cell cycle arrest.	Activation of ER stress, upregulation of p21 and p27, and relocation of focal adhesions in SELENOF-deficient cells [45].	2015
7	Human Lens Epithelial cells	siRNA	Aggravation of the tunicamycin-induced cell apoptosis.	SELENOF knockdown further exacerbated Caspase activation, mitochondrial membrane potential decrease, cytochrome C release, and reactive oxygen species (ROS) generation, with no effect on ER stress [46].	2015
8	Mouse CT26 colon cancer cells	Stably transfecte-d shRNA	Growth and metastasis inhibition in either SELENOF or thioredoxin reductase 1 downregulated colon cancer cells.	Inflammation-related genes regulated by Stat-1, especially interferon-γ-regulated guanylate-binding proteins, were highly elevated in SELENOF-deficient cells, however not in thioredoxin reductase 1-deficient cells. Wnt/β-catenin signaling pathway was upregulated in cells lacking both thioredoxin reductase 1 and SELENOF [47].	2015
9	Mouse	Knockout	Mild splenomegaly and elevated Immunoglobulin Levels without altering immune functions.	Increased secretion of Immunoglobulin M (IgM), delay of ER-to-Golgi glycoprotein transportation [48].	2018
